# A Step Forward in Hypervirulent *Klebsiella pneumoniae* Diagnostics

**DOI:** 10.3201/eid3101.241516

**Published:** 2025-01

**Authors:** Thomas A. Russo, Francois Lebreton, Patrick T. McGann

**Affiliations:** Veterans Administration Western New York Healthcare System, Buffalo, New York, USA (T.A. Russo); University at Buffalo–State University of New York, Buffalo (T.A. Russo); Walter Reed Army Institute of Research, Silver Spring, Maryland, USA (F. Lebreton, P.T. McGann).

**Keywords:** *Klebsiella pneumoniae*, bacteria, diagnostics, antimicrobial resistance

## Abstract

Hypervirulent *Klebsiella pneumoniae* (hvKp) can cause life-threatening infections in healthy community members. HvKp infections often involve multiple sites, some of which are unusual for classical *K. pneumoniae* (cKp) infections, such as the central nervous system, eyes, and fascia. The acquisition of antimicrobial resistance by hvKp has resulted in concerns of an emerging superbug. This concern is magnified by increasing geographic dissemination and healthcare associated infections. Currently, diagnostic testing to differentiate hvKp from cKp is lacking, causing challenges for clinical care, surveillance, and research. Although imperfect, the detection of all 5 of the biomarkers *iucA*, *iroB*, *peg-344*, *rmpA*, and *rmpA2* is the most accurate and pragmatic means to identify hvKp. We propose a working definition for hvKp that will enhance accuracy for diagnosis and surveillance, which will aid in preventing the spread of hvKp.

The increased acquisition of antimicrobial resistance and widening geographic dissemination of hypervirulent *Klebsiella pneumoniae* (hvKp) has magnified concerns (*1*,*2*). HvKp is more virulent than classical *K. pneumoniae* (cKp), which primarily causes healthcare-associated infections (*3*). In contrast, hvKp can also cause organ damage and life-threatening infections in healthy persons and has been found in infection sites that are unusual for classical *K. pneumoniae* (cKp), such as the central nervous system (CNS), eyes, and fascia (*3*,*4*). Recognition of hvKp infection is needed for optimal management. HvKp may infect multiple sites, which may be unrecognized and require source control (*5*,*6*). An awareness of this possibility and liberal imaging is necessary. Further, if unknown sites include the prostate or CNS, the antimicrobial regimen may require adjustment to ensure adequate delivery to those sites (*7*,*8*). Endophthalmitis is a potentially devasting complication of hvKp infection and requires immediate recognition and management with the assistance of an ophthalmologist for both diagnosis and treatment with intravitreal antimicrobial drugs (*8*–*10*). Percutaneous drainage of an abscess is often the preferred method for source control. However, because of the hypermucoviscous nature of hvKp in contrast with cKp, catheter drainage is more challenging because of catheter clogging (*11*). Only using the largest bore catheter possible for drainage will minimize the possibility of failure and the subsequent need for surgical intervention. At present, clinical microbiology laboratories are unable to differentiate between cKp and hvKp. The ability to differentiate between those pathotypes is needed for clinical care, surveillance, and research.

Selected clinical syndromes are highly suggestive of hvKp infection and include community-acquired hepatic abscess, multiple sites of infection, or unusual sites of infection for cKp, such as endophthalmitis, CNS infection, or necrotizing fasciitis in a healthy person. However, infections shared with cKp (e.g., pneumonia, urinary tract infection), healthcare-associated infections, and surveillance studies lacking clinical data demonstrate the need for an accurate test to identify hvKp. The standard is murine infection models; the median lethal dose (LD_50_) of hvKp is 10^1^–10^6^ CFU after challenge, whereas the LD_50_ of cKp is usually >10^7^ CFU (*12*). However, this assessment is reserved for research studies. The commonly used *Galleria mellonella* infection model is unable to differentiate hvKp from cKp (*13*). Although the string test has been used, it lacks optimal sensitivity or specificity (*14*–*16*).

To develop an accurate and pragmatic test, we assembled a hvKp-rich strain cohort by using clinical criteria and a cKp-rich strain cohort by using blood isolates from cKp-rich regions. We established a diagnostic accuracy of >0.95 for the genotypic biomarkers *iucA*, *iroB*, *peg-344*, *rmpA*, and *rmpA2*, differentiating those strain cohorts through phenotypic and genotypic evaluations. We experimentally validated this finding in a mouse systemic infection model where 80/85 (94%) of hvKp strains (defined by an LD_50_ <10^7^ CFU) possessed all 5 of the biomarkers. In contrast, only 1/82 (1.2%) strains with none of the biomarkers had an LD_50_ <10^7^ CFU (*15*).

We identified the strains assessed in this initial study by clinical syndrome, regardless of antimicrobial susceptibility, so the effect of antimicrobial resistance on virulence was unresolved. Therefore, we assembled a cohort of *K. pneumoniae* strains that possessed some combination of *iucA*, *iroB*, *peg-344*, *rmpA*, and *rmpA2* and had acquired resistance, and we categorized them as hvKp or cKp by using a mouse systemic infection model (*17*). The presence of all 5 biomarkers was once again the most accurate (94%) predictor of hvKp, whereas the presence of >4 of biomarkers was most sensitive (100%) (*17*).

Of note, *iucA*, *iroB*, *peg-344*, *rmpA*, and *rmpA2* are linked on canonical hvKp virulence plasmids (*18*), which are shown to be the primary genetic determinant that transforms the baseline virulence potential of cKp strains to the virulence potential observed for hvKp strains (*19*,*20*). A partial set of virulence biomarkers may reflect the presence of an incomplete virulence plasmid, which could affect the hypervirulent phenotype (*20*). In support of this concept, Jaccard and Mash distances in comparison with the canonical pLVPK were the second-most accurate factors for predicting hvKp (*17*). The kleborate virulence score, a commonly used metric calculated in silico from the presence of key loci in genomes (*21*), did not perform as well at predicting phenotypic virulence (*17*).

In clinical microbiology laboratories, assays for *iucA*, *iroB*, *peg-344*, *rmpA*, and *rmpA2* could be readily developed and validated for use by using a PCR platform (*22*). Likewise, genomic analysis could be equally or more insightful, but currently is best suited for research purposes. It is critical to note that both *iutA* and *iroE* can be present on the chromosome and should not be used to differentiate hvKp from cKp (*3*). Although the presence of all 5 biomarkers is most accurate, using >4 markers increased the test sensitivity to 100% compared with 94%, at the cost of decreased specificity (76% using 4 biomarkers, 94% using 5 biomarkers) and accuracy (84% using 4 biomarkers, 92% using 5 biomarkers) (*17*). In addition, occasionally the presence of all 5 biomarkers does not translate to the hvKp pathotype; pathogenesis is a complex, multifactorial process, and in these isolates other requisite factors are absent (*17*). 

In conclusion, we believe that an even more accurate and easily conducted test will be developed in the future. Until then, research studies should not define a strain as hvKp if it does not have all 5 biomarkers without supporting data from an appropriate murine infection model. For the clinical perspective, diagnosing hvKp infection more frequently with less specificity could be considered more pragmatic because the consequences of missing such an infection may be major (e.g., loss of vision). Using the 5 biomarkers discussed to identify hvKp infections will benefit clinicians and patients.
